# Prognostic nutritional index as a potential predictor of prognosis in patients with sepsis: a retrospective cohort study

**DOI:** 10.3389/fnut.2025.1600943

**Published:** 2025-07-01

**Authors:** Mingyuan Pan, Zheng Li, Shanfeng Sheng, Xiao Teng, Yuyang Li

**Affiliations:** ^1^The Second Affiliated Hospital of Guangxi Medical University, Nanning, Guangxi, China; ^2^Wuming Hospital of Guangxi Medical University, Nanning, Guangxi, China

**Keywords:** mortality, prognostic nutritional index, sepsis, prognosis, lymphocyte, albumin

## Abstract

**Background:**

Sepsis patients often have immune dysfunction and malnutrition, which is a high-risk disease for death in critically ill patients. Although various biomarkers can predict the prognosis of sepsis patients, they are cumbersome to implement clinically. This study evaluates the prognostic potential of the Prognostic Nutritional Index (PNI) to fill this gap.

**Methods:**

We conducted a retrospective analysis of data from patients admitted to the Intensive Care Unit (ICU) of Beth Israel Deaconess Medical Center with sepsis between 2008 and 2022. The Prognostic Nutritional Index (PNI) was calculated using the first measurement within 24 h of admission. Kaplan–Meier analysis was used to compare mortality risks among three groups, and a multivariable Cox proportional hazards regression model assessed the link between PNI and mortality risk in sepsis patients. Restricted cubic splines (RCS) explored the potential dose—response relationship between PNI and mortality, and threshold analysis determined the critical threshold of PNI. Receiver operating characteristic (ROC) analysis evaluated the predictive ability, sensitivity, and specificity of LAR for all—cause mortality in patients with liver cirrhosis and sepsis, and calculated the area under the curve (AUC). Finally, subgroup analyses were performed to evaluate the relationship between PNI and prognosis in different populations.

**Results:**

A total of 6,234 patients were included Kaplan—Meier analysis showed that patients with high PNI had lower 14, 28, and 90-day all—cause mortality risks (all log—rank *P* < 0.001). The multivariable Cox proportional hazards model indicated that high PNI was independently associated with 14, 28, and 90-day all—cause mortality, with HRs of 0.62, 0.56, and 0.59 (all *P* < 0.0001), before and after adjusting for confounders RCS analysis revealed a non-linear link between PNI and short—and medium—term all—cause mortality in sepsis patients. A two—segment Cox proportional hazards model identified inflection points at 11.6 for 14-day, 11.2 for 28-day, and 11.2 for 90-day all-cause mortality ROC analysis showed PNI has lower predictive value for sepsis prognosis than sequential organ failure assessment and acute physiology and chronic health evaluation, yet it can enhance their predictive power Subgroup analyses found no significant interaction between PNI and specific subgroups.

**Conclusion:**

There is a significant association between short-term and medium—term all—cause mortality in sepsis patients and PNI, indicating that PNI can be a valuable indicator for predicting in—hospital and ICU mortality risk.

## 1 Introduction

Sepsis is defined as life-threatening organ dysfunction caused by a dysregulated host response to infection. It is one of the common reasons for sending critically ill patients to the intensive care unit and a major cause of death in such patients (1). This dysregulated response to infection can lead to cellular dysfunction and ultimately organ dysfunction. According to relevant studies, the mortality rate of sepsis can be as high as 40% (2, 3).

Albumin is a good indicator of nutritional status and is closely related to the prognosis of sepsis patients (4). However, baseline nutritional status or chronic inflammatory diseases can affect albumin levels, limiting its use as a single prognostic indicator. Peripheral blood lymphocyte levels are key immune indicators in patients with infection, and changes in their number and function can reflect the body’s immune status (5, 6). For example, in sepsis, patients experience increased lymphocyte apoptosis, leading to reduced immune cell numbers and decreased function. However, severe infections and certain medications can cause abnormal lymphocyte levels, limiting the prediction of patient prognosis based solely on lymphocyte levels (7). Therefore, identifying indicators to assess the prognosis of sepsis patients is crucial for timely recognition and intervention.

Recently, the Prognostic Nutritional Index (PNI), calculated from albumin and lymphocyte levels, has shown potential as a predictor of mortality in various conditions, including cancer, cardiovascular disease, liver disease, and chronic kidney disease (8–12). However, the mid-term prognostic value of PNI in sepsis patients has not been reported.

In this study, we aim to retrospectively analyze the clinical data of critically ill sepsis patients to explore the potential of PNI as a mid-term prognostic tool, with the goal of aiding early clinical recognition and improving outcomes.

## 2 Materials and methods

### 2.1 Data source

We retrospectively analyzed clinical data of sepsis patients extracted from the Medical Information Mart for Intensive Care IV (MIMIC-IV) database (version 3.1) between 2008 and 2022 (13). The MIMIC-IV database, developed by the Massachusetts Institute of Technology’s Computational Physiology Laboratory, includes records of patients admitted to Beth Israel Deaconess Medical Center. Our research team completed the Collaborative Institutional Training Initiative (CITI) course, passed the “Conflict of Interest” and “Research Data or Specimens Only” exams, and obtained access to the MIMIC-IV database (version 3.1). The study aimed to explore the potential of PNI as a prognostic tool to aid early clinical recognition and improve outcomes.

### 2.2 Study population

**FIGURE 1:**
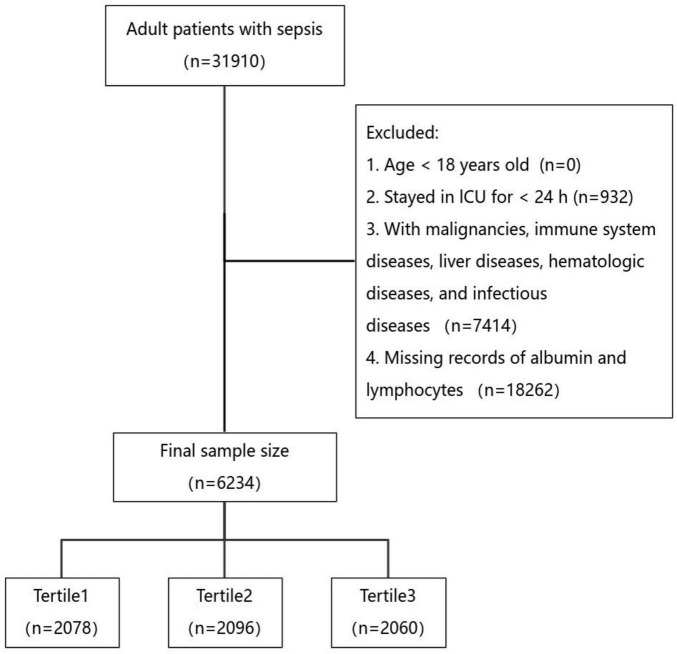
Flowchart for participants from MIMIV-IV (v 3.1).

Patients were classified as having sepsis according to the Sepsis 3.0 definition if they had suspected infection and a Sequential Organ Failure Assessment (SOFA) score ≥ 2 (1). Strict exclusion criteria were applied to ensure robust results, including patients under 18 years of age at first admission, patients with ICU stays less than 24 h, patients with conditions affecting albumin and lymphocyte levels, and patients without albumin and lymphocyte records within the first 24 h of admission. Only the first admission data were included for patients with multiple ICU stays. Ultimately, 6,234 patients were included in the study (Figure 1).

### 2.3 Data extraction

Data extraction was performed using PostgreSQL software (version 13.7.2) and Navicat Premium software (version 16) with the aid of Structured Query Language (SQL). Information on demographic characteristics, vital signs, comorbidities, laboratory data, microbiological data, clinical treatments, survival status, and severity scores was extracted.

### 2.4 Handling of outliers and missing values

Outliers were handled using the winsor2 command in STATA with 1 and 99% cutoff points. Missing values were imputed using multiple imputation, excluding variables with over 10% missing data and imputing the remaining using this method.

### 2.5 Grouping and clinical outcomes

PNI was calculated as: PNI = albumin (g/dL) + 5 × absolute lymphocyte count (× 10^9^/L) (14). Patients were divided into three groups by tertiles. The primary endpoint was 90-day all—cause mortality, with secondary endpoints at 14 and 28 days.

### 2.6 Ethical statement

This study adhered to the Declaration of Helsinki. As it used de-identified data from MIMIC-IV (version 3.1), the Beth Israel Deaconess Medical Center’s ethics review committee waived the requirement for informed consent. No formal ethical approval or patient consent was needed due to the use of de-identified data.

### 2.7 Statistical analysis

Continuous variables are presented as median (IQR). The Mann-Whitney U or Kruskal-Wallis test was used for group comparisons. Categorical variables are summarized as frequency (percentage), with group comparisons done via chi—square or Fisher’s exact tests.

Patients were divided into three groups based on PNI tertiles. Kaplan–Meier curves were plotted to analyze survival trajectories for primary and secondary endpoints, with log—rank tests assessing group differences. Univariate and multivariate Cox proportional hazards models evaluated the association between PNI (as a categorical variable) and 14, 28, and 90-day all—cause mortality. The multivariate models were adjusted for potential confounding factors such as age, gender, race, SOFA score, hematocrit, platelets, anion gap, calcium, sodium, lactate, acute kidney injury, chronic kidney disease, myocardial infarction, continuous renal replacement therapy, mechanical ventilation, vasoactive drugs, albumin, immunosuppressants, and corticosteroids.

Restrictive cubic spline (RCS) analysis based on Cox regression models was performed to explore the potential non-linear relationship between PNI levels and mortality. A two—segment Cox proportional hazards model was used to determine threshold effects and identify inflection points.

Receiver operating characteristic (ROC) analysis was conducted to evaluate the predictive ability of PNI, SOFA, APACHE II, and their combinations for all—cause mortality at different time points, and the area under the curve (AUC) was calculated.

Subgroup analyses were performed to assess the consistency of the association between PNI levels and mortality across predefined subgroups, including age, sex, acute kidney injury, chronic kidney disease, myocardial infarction, albumin treatment, immunosuppressive treatment, and corticosteroid therapy.

All analyses were performed using the Decision Linnc (Decision Linnc Core Team 2023) analytical platform, a comprehensive platform that combines various programming environments and enables data processing and analysis. Hang Zhou, CHN. Retrieved from https://www.statsape.com/. All the statistical tests were two-tailed, with a significance threshold of *P* < 0.05.

## 3 Results

### 3.1 Baseline characteristics comparison of sepsis patients in three groups

As shown in Table 1, this study included 6,234 patients, with a mean age of 66.00 (18.00–90.00) years, and 2,627 (42.14%) were female. ICU mortality was 14.87%, in—hospital mortality 23.36%, 14-day all—cause mortality 15.86%, 28-day 21.05%, and 90-day 23.71%. Patients were divided into three groups by PNI tertiles: T1 (low), T2 (medium), and T3 (high). Baseline characteristics differed significantly across groups. The high—PNI group had higher rates of hypertension, chronic kidney disease, and pneumonia, with a higher likelihood of Acinetobacter baumannii and gram—positive bacterial infections. The low—PNI group had more heart failure and myocardial infarction cases and was more prone to Candida albicans and gram—negative bacterial infections. Additionally, the high—PNI group had lower SOFA and APACHE II scores, received more albumin therapy, and less immunosuppressive therapy. Notably, the high—PNI group had lower mortality rates across all time endpoints compared to other groups (Table 1).

**TABLE 1:** Baseline characteristics.

Variable	Overall *N* = 6,234	T1 = 2,078	T2 = 2,096	T3 = 2,060	*P*- value
PNI	7.90 (1.30–1,630.35)	5.00 (1.30–6.45)	7.90 (6.50–9.75)	12.65 (9.80–1,630.35)	<0.001
**Demographics**					
Age,year	66.00 (18.00–90.00)	68.00 (18.00–90.00)	65.00 (18.00–90.00)	65.00 (19.00–90.00)	<0.001
Gender, n(p%)					0.072
Male	3,607.00 (57.86%)	1,244.00 (59.87%)	1,197.00 (57.11%)	1,166.00 (56.60%)	
Female	2,627.00 (42.14%)	834.00 (40.13%)	899.00 (42.89%)	894.00 (43.40%)	
Race,n(p%)					0.243
Asian	198.00 (3.17%)	79.00 (3.80%)	58.00 (2.76%)	61.00 (2.95%)	
White	3,533.00 (56.67%)	1,230.00 (59.19%)	1,162.00 (55.44%)	1,141.00 (55.39%)	
Black	601.00 (9.64%)	178.00 (8.57%)	211.00 (10.07%)	212.00 (10.29%)	
Other	1902(30.51%)	591.00 (28.44%)	665.00 (31.73%)	646.00 (31.36%)	
**Comorbidities**					
Hypertension,n(p%)	2,053.00 (32.93%)	627.00 (30.17%)	693.00 (33.06%)	733.00 (35.58%)	0.001
Acute Kidney Injury, n (%)	3,799.00 (60.94%)	1,379.00 (66.36%)	1,249.00 (59.59%)	1,171.00 (56.84%)	<0.001
Cerebrovascular Disease, n (%)	2,742.00 (43.98%)	934.00 (44.95%)	921.00 (43.94%)	887.00 (43.06%)	0.118
Chronic Kidney Disease, n (%)	359.00 (5.76%)	102.00 (4.91%)	127.00 (6.06%)	130.00 (6.31%)	0.015
Type 2 Diabetes, n (%)	1,416.00 (22.71%)	515.00 (24.78%)	466.00 (22.23%)	435.00 (21.12%)	0.499
Type 1 Diabetes, n (%)	1,888.00 (30.29%)	612.00 (29.45%)	640.00 (30.53%)	636.00 (30.87%)	0.582
Heart Failure, n (%)	91.00 (1.46%)	35.00 (1.68%)	31.00 (1.48%)	25.00 (1.21%)	0.498
Myocardial infarction, n (%)	1,916.00 (30.73%)	647.00 (31.14%)	656.00 (31.30%)	613.00 (29.76%)	0.007
Pneumonia, n (%)	1,000.00 (16.04%)	307.00 (14.77%)	320.00 (15.27%)	373.00 (18.11%)	0.472
**Scores**					
SOFA score	7.00 (0.00–23.00)	7.00 (0.00–23.00)	6.00 (0.00–21.00)	6.00 (0.00–22.00)	<0.001
APACHE II Score	20.00 (1.00–53.00)	22.00 (3.00–52.00)	20.00 (1.00–49.00)	19.00 (1.00–53.00)	<0.001
**Vital signs**					
Heart rate, bpm	92.00 (0.00–191.00)	95.00 (0.00–191.00)	91.00 (0.00–186.00)	90.00 (28.00–182.00)	<0.001
Systolic blood pressure, mmHg	118.00 (35.00–248.00)	115.00 (35.00–248.00)	118.99 (39.00–226.00)	118.00 (36.00–210.00)	<0.001
Diastolic blood pressure, mmHg	68.00 (11.00–70,130.00)	67.00 (11.00–70,130.00)	69.00 (14.00–168.00)	68.00 (20.00–190.00)	<0.001
Mean arterial pressure, mmHg	81.00 (0.00–140,119.00)	80.00 (17.00–140,119.00)	82.00 (12.00–936.00)	81.00 (0.00–6,116.00)	<0.001
Respiratory rate, bpm	20.00 (0.00–91.00)	21.00 (0.00–91.00)	20.00 (0.00–61.00)	20.00 (0.00–57.00)	<0.001
Oxygen saturation,%	97.00 (19.00–9,819.00)	97.00 (60.00–963.00)	97.00 (55.00–100.00)	98.00 (19.00–9,819.00)	<0.001
Temperature,°F	98.40 (0.00–104.40)	98.30 (0.00–104.40)	98.40 (34.10–103.30)	98.35 (34.80–103.70)	0.571
**Laboratory parameters**					
White blood cells,109/L	12.40 (0.10–406.30)	10.60 (0.10–406.30)	12.50 (1.00–79.70)	13.90 (1.80–365.70)	<0.001
Red blood cells,109/L	3.44 (1.07–7.21)	3.27 (1.13–6.82)	3.51 (1.07–6.95)	3.59 (1.18–7.21)	<0.001
Hemoglobin, g/L	10.20 (3.50–21.00)	9.70 (3.50–21.00)	10.40 (3.60–19.70)	10.50 (3.60–18.50)	<0.001
Platelets, 109/L	178.00 (6.00–1,254.00)	152.00 (6.00–1,254.00)	185.00 (10.00–819.00)	195.00 (6.00–1,028.00)	<0.001
Neutrophil cells,109/L	9.97 (0.00–117.83)	9.06 (0.00–117.83)	9.82 (0.00–70.98)	10.92 (0.00–97.52)	<0.001
Lymphocyte cells, 109/L	0.99 (0.00–325.69)	0.43 (0.00–1.05)	0.99 (0.42–1.65)	1.91 (0.96–325.69)	<0.001
Hematocrit,%	31.70 (11.30–66.40)	30.10 (11.30–66.40)	32.10 (11.50–62.80)	32.70 (11.90–55.40)	<0.001
Erythrocyte distribution width,%	14.80 (11.10–33.10)	15.40 (11.50–29.00)	14.70 (11.10–28.10)	14.60 (11.10–33.10)	<0.001
Total bilirubin, mg/dL	0.70 (0.10–62.80)	0.80 (0.10–49.90)	0.70 (0.10–62.80)	0.70 (0.10–51.20)	<0.001
Alanine aminotransferase, U/L	31.00 (3.00–15,018.00)	32.00 (4.00–15,018.00)	31.00 (3.00–13,330.00)	30.00 (3.00–6,627.00)	0.181
Aspartate aminotransferase,U/L	46.00 (5.00–28,275.00)	47.00 (5.00–28,275.00)	47.00 (5.00–27,004.00)	45.00 (5.00–14,244.00)	0.946
Albumin, g/dL	2.90 (0.30–5.50)	2.70 (0.30–4.90)	3.00 (0.60–5.00)	3.10 (1.00–5.50)	<0.001
Creatinine, mg/dL	1.20 (0.00–32.00)	1.30 (0.20–19.10)	1.20 (0.20–32.00)	1.10 (0.00–17.30)	<0.001
Blood urea nitrogen, mg/dL	23.00 (1.00–213.00)	28.00 (2.00–212.00)	22.00 (1.00–213.00)	20.00 (1.00–184.00)	<0.001
Anion gap, mmol/L	15.00 (−8.00–55.00)	15.00 (−8.00–45.00)	15.00 (1.00–55.00)	14.00 (4.00–53.00)	< 0.001
Calcium, mmol/L	8.30 (1.70–18.90)	8.10 (1.70–18.90)	8.30 (4.40–14.20)	8.35 (4.20–14.50)	<0.001
Potassium, mmol/L	4.20 (1.90–9.80)	4.20 (2.10–9.80)	4.20 (1.90–9.00)	4.20 (1.90–9.70)	0.785
Sodium, mmol/L	138.00 (100.00–178.00)	137.00 (103.00–165.00)	138.00 (100.00–170.00)	138.00 (106.00–178.00)	<0.001
Lactate, mmol/L	2.00 (0.30–29.20)	2.10 (0.30–22.00)	1.90 (0.40–29.20)	2.00 (0.50–22.00)	0.012
Glucose, mg/dL	134.00 (19.00–2,044.00)	136.00 (19.00–1,260.00)	134.50 (23.00–2,044.00)	132.00 (19.00–1,205.00)	0.354
International normalized ratio	1.40 (0.80–14.70)	1.40 (0.90–13.10)	1.30 (0.80–14.70)	1.30 (0.80–13.20)	<0.001
Prothrombin time,s	14.80 (8.30–150.00)	15.20 (9.50–150.00)	14.60 (8.30–123.90)	14.55 (8.80–150.00)	<0.001
Activated partial thromboplastin time,s	31.30 (17.80–150.00)	31.90 (18.10–150.00)	31.10 (18.50–150.00)	31.10 (17.80–150.00)	0.118
PH	7.35 (6.53–7.78)	7.34 (6.69–7.78)	7.35 (6.78–7.65)	7.36 (6.53–7.64)	<0.001
PaO2	79.00 (14.00–681.00)	67.00 (15.00–681.00)	80.50 (15.00–612.00)	89.00 (14.00–600.00)	<0.001
PCO2	42.00 (9.00–148.00)	42.00 (14.00–112.00)	42.00 (16.00–130.00)	42.00 (9.00–148.00)	0.367
**Microbiome**					0.126
Klebsiella pneumoniae, n (%)	2069(33.19%)	659(31.71%)	729(34.78%)	681(33.06%)	
S. aureus positive, n (%)	597(9.98%)	237(11.40%)	144(6.87%)	216(10.48%)	
Other staphylococci, n (%)	62(0.99%)	10(0.48%)	21 (1.00%)	31(1.50%)	
Acinetobacter baumannii, n (%)	329(5.28%)	93(4.47%)	113(5.39%)	123(5.97%)	
Candida albicans, n (%)	885(14.20%)	453(21.80%)	236(11.26%)	196(9.51%)	
Gram positive bacteria, n (%)	875(14.04%)	309(14.87%)	246(11.74%)	320(15.53%)	
Gram negative bacteria, n (%)	52(0.83%)	31(1.49%)	10 (0.48%)	11(0.53%)	
**Treatments**					
Continuous renal replacement therapy, n (%)	895.00 (14.36%)	342.00 (16.46%)	296.00 (14.12%)	257.00 (12.48%)	0.001
Ventilation, n (%)	5,657.00 (90.74%)	1,868.00 (89.89%)	1,890.00 (90.17%)	1,899.00 (92.18%)	0.021
Antibiotics, n (%)	6,200.00 (99.45%)	2,074.00 (99.81%)	2,083.00 (99.38%)	2,043.00 (99.17%)	0.019
Vasopressors, n (%)	4,745.00 (76.11%)	1,598.00 (76.90%)	1,552.00 (74.05%)	1,595.00 (77.43%)	0.022
Albumin, n (p%)	1,239.00 (19.87%)	408.00 (19.63%)	369.00 (17.60%)	462.00 (22.35%)	<0.001
Immunos, n (p%)	149.00 (2.39%)	90.00 (4.33%)	38.00 (1.81%)	21.00 (1.02%)	<0.001
Glucocorticoids, n (p%)	1,702.00 (27.30%)	671.00 (32.29%)	524.00 (25.00%)	507.00 (24.61%)	<0.001
**Clinical outcomes**					
ICU mortality, n (%)	927.00 (14.87%)	384.00 (18.48%)	307.00 (14.65%)	236.00 (11.46%)	<0.001
In-hospital mortality, n (%)	1,456.00 (23.36%)	617.00 (29.69%)	470.00 (22.42%)	369.00 (17.91%)	<0.001
14-day all-cause mortality, n (%)	989.00 (15.86%)	438.00 (21.08%)	308.00 (14.69%)	243.00 (11.80%)	<0.001
28-day all-cause mortality, n (%)	1,312.00 (21.05%)	573.00 (27.57%)	420.00 (20.04%)	319.00 (15.49%)	<0.001
90-day all-cause mortality, n (%)	1,478.00 (23.71%)	630.00 (30.32%)	478.00 (22.81%)	370.00 (17.96%)	<0.001

### 3.2 Kaplan–Meier survival curves

**FIGURE 2:**
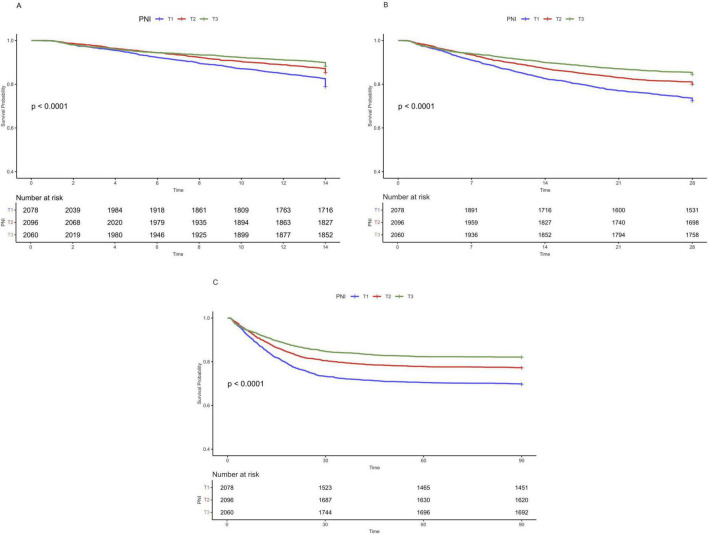
Kaplan-Meier curves and cumulative incidence of 14-day **(A)**, 28-day **(B)**, and 90-day **(C)** all-cause mortality stratified by PNI groups.

A total of 989 patients died within 14 days, 1,312 within 28 days, and 1,478 within 90 days. Kaplan–Meier survival curves showed that patients with higher PNI had lower 14, 28, and 90-day all—cause mortality (Figure 2, all log-rank *P* < 0.001). [Kaplan—Meier curves and cumulative incidence of 14-day (A), 28-day (B), and 90-day (C) all-cause mortality stratified by PNI groups].

### 3.3 Relationship between PNI levels and clinical outcomes

A multivariable Cox regression model was constructed to analyze the association between PNI levels and 14, 28, and 90-day all—cause mortality in sepsis patients. In the unadjusted model (Model 1), higher PNI was significantly associated with lower mortality risk: HR = 0.53 (95% CI:0.46–0.63, *P* < 0.0001) for 14-day, HR = 0.51 (95% CI:0.45–0.59, *P* < 0.0001) for 28-day, and HR = 0.54 (95% CI:0.47–0.61, *P* < 0.0001) for 90-day mortality. Model 2 adjusted for age, sex, and race, and also showed lower mortality risk with higher PNI: HR = 0.55 (95% CI:0.47–0.64, *P* < 0.0001) for 14-day, HR = 0.52 (95% CI:0.45–0.60, *P* < 0.0001) for 28-day, and HR = 0.54 (95% CI:0.48–0.62, *P* < 0.0001) for 90-day mortality. Model 3 further adjusted for potential confounders, including SOFA score, hematocrit, platelets, anion gap, calcium, sodium, lactate, acute kidney injury, chronic kidney disease, myocardial infarction, renal replacement therapy, mechanical ventilation, vasoactive drugs, albumin, immunosuppressants, and corticosteroids. Higher PNI levels remained significantly associated with lower all—cause mortality (Table 2).

**TABLE 2:** Cox regression model.

	Model 1		Model 2		Model 3	
	HR (95% CI)	*P*-value	HR (95% CI)	*P*-value	HR (95% CI)	*P*-value
**14-day all-cause mortality**						
PNI	0.93(0.91–0.94)	<0.0001	0.93(0.91–0.95)	<0.0001	0.95(0.93–0.97)	<0.0001
T1	Reference		Reference		Reference	
T2	0.68(0.58–0.78)	<0.0001	0.68(0.58–0.78)	<0.0001	0.81(0.70–0.94)	0.007
T3	0.53(0.46–0.63)	<0.0001	0.55(0.45–0.64)	<0.0001	0.62(0.53–0.73)	<0.0001
P for trend	<0.0001		<0.0001		<0.0001	
**28-day all-cause mortality**						
PNI	0.92(0.91–0.94)	<0.0001	0.92(0.91–0.94)	<0.0001	0.94(0.92–0.95)	<0.0001
T1	Reference		Reference		Reference	
T2	0.69(0.61–0.79)	<0.0001	0.69(0.61–0.79)	<0.0001	0.82(0.72–0.93)	0.002
T3	0.51(0.45–0.59)	<0.0001	0.52(0.46–0.60)	<0.0001	0.56(0.50–0.67)	<0.0001
P for trend	<0.0001		<0.0001		<0.0001	
**90-day all-cause mortality**						
PNI	0.93(0.91–0.94)	<0.0001	0.93(0.91–0.94)	<0.0001	0.94(0.93–0.96)	<0.0001
T1	Reference		Reference		Reference	
T2	0.71(0.63–0.80)	<0.0001	0.71(0.63–0.80)	<0.0001	0.82(0.73–0.92)	0.001
T3	0.54(0.47–0.61)	<0.0001	0.54(0.48–0.62)	<0.0001	0.59(0.52–0.68)	<0.0001
P for trend	<0.0001		<0.0001		<0.0001	

Model 1: unadjusted, Model 2: adjusted age, gender, and ethnicity, Model 3: adjusted age, gender, ethnicity, sequential organ failure assessment, hematocrit, platelets, anion gap, calcium, sodium, lactate, acute kidney injury, chronic kidney disease, myocardial infarction, continuous renal replacement therapy, mechanical ventilation, vasopressors,albumin, immunos and glucocorticoids.

### 3.4 Detection of non-linear relationships

**FIGURE 3:**
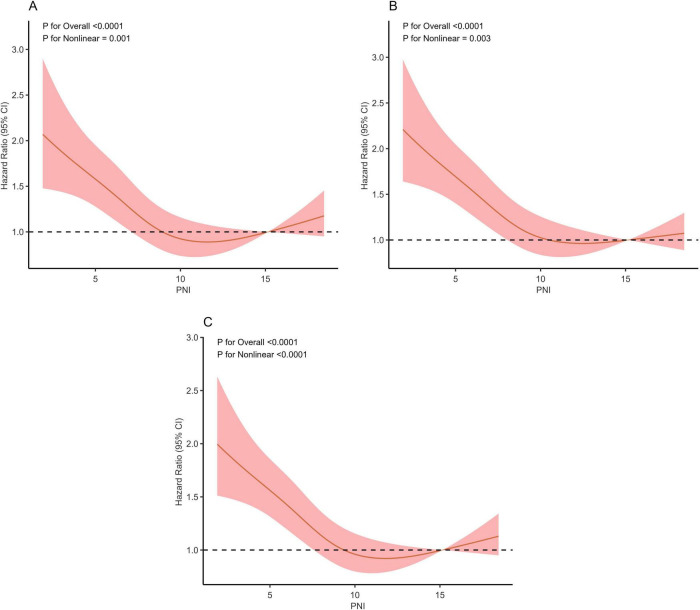
Restricted cubic spline regression analysis of PNI with 14-day **(A)**, 28-day **(B)**, and 90-day **(C)** all-cause mortality.

RCS analysis showed a non-linear relationship between PNI and 14, 28, and 90-day all—cause mortality (Figure 3, all P for non-linearity < 0.01). A two-segment Cox proportional hazards model identified inflection points at 14 days (11.6), 28 days (11.2), and 90 days (11.2). Below these thresholds, each 1-unit PNI increase was linked to a 9% drop in mortality risk, with HRs of 0.91 (95% CI: 0.88–0.94) for 14-day, 0.91 (95% CI: 0.89–0.93) for 28-day, and 0.91 (95% CI: 0.89–0.93) for 90-day mortality, indicating a negative PNI-mortality risk correlation. Conversely, when PNI was above these thresholds, the mortality risk rose, with HRs of 1.08 (95% CI: 1.01–1.15) for 14-day and 1.05 (95% CI: 1.00–1.10) for 90-day mortality. All associations were statistically significant (*p* < 0.001 by likelihood ratio test) (Table 3).

**TABLE 3:** Two-piecewise Cox proportional model.

	Adjusted HR (95% CI)	*p*-value
**14-day all-cause mortality**		
Inflection point	11.6	
PNI < 11.6	0.91(0.88–0.94)	<0.001
PNI ≥ 11.6	1.08(1.01–1.15)	0.026
p for likelihood ratio test		<0.001
**28-day all-cause mortality**		
Inflection point	11.2	
PNI < 11.2	0.91(0.89–0.93)	<0.001
PNI ≥ 11.2	1.04(0.99–1.09)	0.182
p for likelihood ratio test		<0.001
**90-day all-cause mortality**		
Inflection point	11.2	
PNI < 11.2	0.91(0.89–0.93)	<0.001
PNI ≥ 11.2	1.05(1.00–1.10)	0.036
p for likelihood ratio test		<0.001

Adjusted models adjusted for age, gender, ethnicity, sequential organ failure assessment, hematocrit, platelets, anion gap, calcium, sodium, lactate, acute kidney injury, chronic kidney disease, myocardial infarction, continuous renal replacement therapy, mechanical ventilation, vasopressors,albumin, immunos and glucocorticoids

### 3.5 Predictive efficacy of PNI for all-cause mortality in in sepsis patients

**FIGURE 4:**
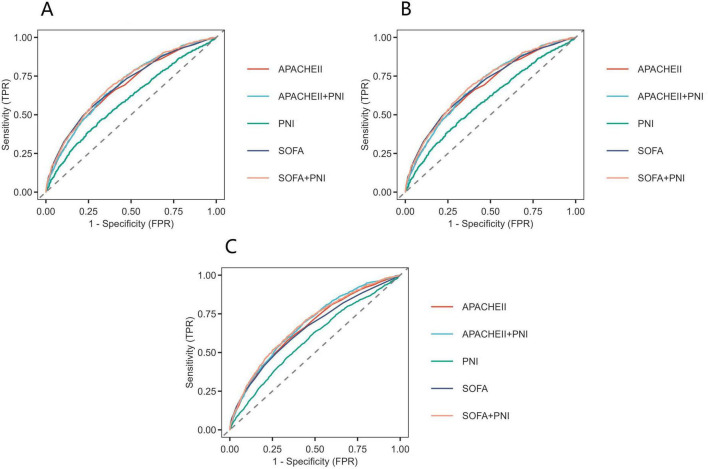
Receiver operating characteristic curves assesses the predictive capability of the PNI for 14-day **(A)**, 28-day **(B)**, and 90-day **(C)** all-cause mortality.

The predictive value of PNI for all—cause mortality in sepsis patients was assessed by plotting ROC curves for PNI, SOFA, APACHE II, and their combinations, analyzing their predictive power for 14, 28, and 90-day all-cause mortality (Figures 4A–C). For 90-day mortality, the AUC for PNI was 0.59 (95% CI: 0.57–0.61), for SOFA 0.65 (95% CI: 0.64–0.67), for SOFA plus PNI 0.68 (95% CI: 0.67–0.70), for APACHE II 0.66 (95% CI: 0.65–0.68), and for APACHE II plus PNI 0.68 (95% CI: 0.67–0.70). While PNI alone had a lower predictive value than SOFA and APACHE II, it could enhance their predictive power when used in combination. Detailed results are presented in Figure 4 and Table 4.

**TABLE 4:** Information of ROC curves in figure.

	AUC	95% CI	Threshold	Sensitivity	Septicity	*p*-value
**14-day all-cause mortality**						
PNI	0.61	0.59–0.63	1.22	0.69	0.50	
SOFA	0.68	0.67–0.70	1.27	0.72	0.55	Reference
SOFA + PNI	0.70	0.68–0.72	1.12	0.64	0.66	<0.001
APACHE II	0.68	0.66–0.70	1.12	0.65	0.62	Reference
APACHE II + PNI	0.70	0.68–0.72	1.18	0.66	0.63	<0.001
**28-day all-cause mortality**						
PNI	0.60	0.58–0.62	1.21	0.68	0.48	
SOFA	0.66	0.65–0.68	1.24	0.73	0.51	Reference
SOFA + PNI	0.70	0.68–0.72	1.10	0.64	0.66	<0.001
APACHE II	0.67	0.66–0.69	1.11	0.66	0.59	Reference
APACHE II + PNI	0.69	0.67–0.71	0.99	0.56	0.72	<0.001
**90-day all-cause mortality**						
PNI	0.59	0.57–0.61	1.03	0.51	0.63	
SOFA	0.65	0.64–0.67	1.07	0.64	0.58	Reference
SOFA + PNI	0.68	0.67–0.70	1.03	0.60	0.66	<0.001
APACHE II	0.66	0.65–0.68	1.11	0.66	0.58	Reference
APACHE II + PNI	0.68	0.67–0.70	1.00	0.57	0.69	<0.001

### 3.6 Subgroup analysis

**FIGURE 5:**
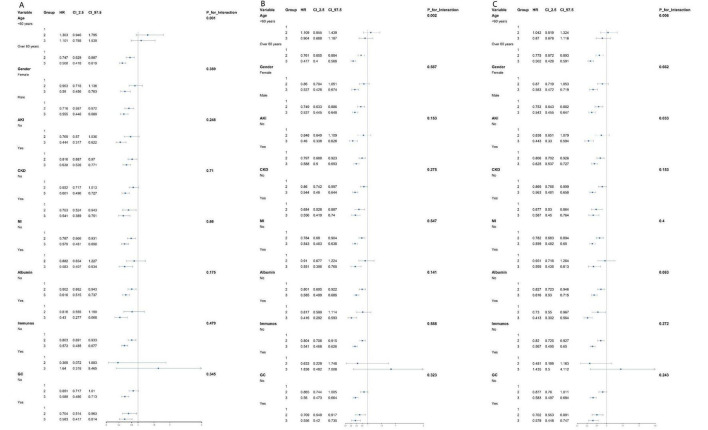
Forest plots of stratified analyses of PNI and 14-day **(A)**, 28-day **(B)**, and 90-day **(C)** all-cause mortality in patients.

We further explored the link between PNI and the risk of 14, 28, and 90-day all—cause mortality across different populations. Forest-plot analysis showed no significant PNI-subgroup interactions when stratifying by age, sex, acute kidney injury, chronic kidney disease, myocardial infarction, albumin, immunosuppressant, and corticosteroid use (all *P*-values for interaction > 0.05) (Figure 5).

## 4 Discussion

To our knowledge, this is the first study to evaluate the relationship between PNI and mid-term prognosis in sepsis patients. Our results indicate a significant association between PNI levels and 14, 28, and 90-day all—cause mortality in this population, even after adjusting for potential confounders.

Sepsis is a systemic inflammatory response syndrome caused by infection, characterized by an excessive host response leading to systemic inflammation and immune dysfunction (1). This excessive response triggers systemic inflammation and immune dysfunction, and is often accompanied by malnutrition and immune disorders. During the early stages of sepsis, the immune system is activated, releasing a large number of pro-inflammatory cytokines. These cytokines can cause tissue damage and organ dysfunction (7), leading to high mortality and readmission rates among sepsis patients (8–10). Therefore, identifying a clinical biomarker that is easily obtainable and has high predictive value for early intervention in sepsis patients is crucial for improving their prognosis.

Albumin is a key indicator of nutritional status and also reflects the severity of inflammation (4). However, albumin levels can be influenced by various factors such as liver dysfunction and chronic inflammation, which limit the predictive value of albumin alone (15–18). Peripheral blood lymphocytes are important indicators of immune function and serve as a key component of the indirect markers of immune dysfunction in sepsis (5, 19). Nevertheless, in sepsis patients with concurrent infections or abnormal immune system activation, peripheral blood lymphocytes may increase (20–22). This reduces the reliability of using lymphocytes alone to predict patient outcomes.

The PNI, calculated based on serum albumin and lymphocyte counts, helps to reduce the confounding effects of nutrition and inflammation on albumin interpretation. It comprehensively evaluates a patient’s nutritional and immune status, thereby predicting their prognosis. Generally, a higher PNI value indicates better nutritional status and immune function, and a lower risk of death.

Previous studies have confirmed the prognostic value of PNI in various diseases, such as liver cirrhosis, heart failure, stroke-related pneumonia, and tumors (23–26). In critically ill patients (27), PNI can effectively identify patients at high nutritional risk, who often have poor clinical outcomes, including higher mortality, longer mechanical ventilation time, and longer ICU stay. Moon Seong Baek et al. (28) further pointed out that elderly sepsis patients with low PNI levels have higher mortality rates.

### 4.1 This study further confirms the prognostic significance of PNI in sepsis patients

We collected data from 6,234 sepsis patients and compared the baseline information of patients in three PNI groups. Multivariable Cox regression analysis of mortality outcomes showed that PNI was an independent risk factor for this patient population. Our results are consistent with previous studies, indicating that patients with lower PNI may have inadequate nutrition, poor infection control, and immune imbalance, which can lead to reduced albumin and lymphocyte levels. Conversely, patients in the high PNI group have improved nutritional status and organ function due to controlled infections and nutritional support. This study also reveals a non-linear relationship between PNI and mortality risk in sepsis patients, consistent with existing evidence. This further emphasizes the prognostic importance of PNI in this population and identifies specific risk thresholds.

Scoring systems like SOFA and APACHE II have long been key tools for assessing disease severity and organ dysfunction in critically ill patients (29–31). Our study shows that PNI has relatively good predictive power and can enhance the ability of these two scoring systems to predict the prognosis of sepsis patients.

Clinically, based on PNI assessment, early nutritional intervention should be implemented for high-risk critically ill patients. Appropriate early nutritional support improves status, reduces complications, and enhances survival. Individualized nutritional management plans, based on PNI and other assessments, are necessary due to varying baseline nutrition, disease types, and severities. Dynamic monitoring of PNI and adjusting support ensures optimal outcomes.

A major strength of this study is establishing PNI as an independent predictor of short-term and long-term all-cause mortality in sepsis patients. The diverse population data in the MIMIC-IV database (version 3.1) allowed robust statistical adjustments to mitigate confounding effects.

Despite these strengths, our study has limitations. As a single-center retrospective study, our findings may lack generalizability and require prospective validation. We only assessed initial PNI levels at admission and did not evaluate dynamic changes over time. Future studies on dynamic PNI measurements are needed to further clarify its clinical utility. Additionally, while we used measurements within the first 24 h to minimize the impact of interventions, we could not confirm if patients received nutritional or immune interventions before albumin and lymphocyte measurements due to MIMIC-IV limitations. Future research should explore how such interventions affect albumin, lymphocyte levels, and PNI predictive ability. Lastly, despite multivariable adjustments and subgroup analyses, other unmeasured confounders may influence results.

## 5 Conclusion

This study shows a significant association between short-term and medium-term all-cause mortality in sepsis patients and PNI, indicating PNI can be a valuable indicator for predicting in-hospital and ICU mortality risk. Low PNI levels correlate with higher mortality risk and poorer prognosis, while increasing PNI may improve clinical outcomes. Clinically, monitoring PNI in sepsis patients is essential, with timely nutritional support and other interventions to optimize prognosis.

## Data Availability

The datasets presented in this study can be found in online repositories. The names of the repository/repositories and accession number(s) can be found at: mimic.physionet.org/.
